# PGE_2_ is a direct and robust mediator of anion/fluid secretion by human intestinal epithelial cells

**DOI:** 10.1038/srep36795

**Published:** 2016-11-09

**Authors:** Satoru Fujii, Kohei Suzuki, Ami Kawamoto, Fumiaki Ishibashi, Toru Nakata, Tatsuro Murano, Go Ito, Hiromichi Shimizu, Tomohiro Mizutani, Shigeru Oshima, Kiichiro Tsuchiya, Tetsuya Nakamura, Akihiro Araki, Kazuo Ohtsuka, Ryuichi Okamoto, Mamoru Watanabe

**Affiliations:** 1 Department of Gastroenterology and Hepatology, Graduate School, Tokyo Medical and Dental University, Tokyo, Japan; 2 Institute of Clinical Molecular Biology, Christian-Albrechts-University Kiel, Kiel, Germany; 3 Department of Medicine, University of California, San Francisco, San Francisco, California, USA; 4 Department of Advanced Therapeutics in GI Diseases, Graduate School, Tokyo Medical and Dental University, Tokyo, Japan; 5 Center for Stem Cell and Regenerative Medicine, Graduate School, Tokyo Medical and Dental University, Tokyo, Japan

## Abstract

Intestinal epithelial cells (IECs) play an indispensable role in maintaining body fluid balance partly through their ability to regulate anion/fluid secretion. Yet in various inflammatory gastrointestinal diseases, over-secretion of anions results in symptoms such as severe diarrhoea. Endogenous mediators, such as vasoactive intestinal peptide or prostaglandin E_2_ (PGE_2_), regulate intestinal anion/fluid secretion, but their direct effect on purified human IECs has never been described in detail. Based on a previously described intestinal organoid swelling model, we established a 3D-scanner-assisted quantification method to evaluate the anion/fluid secretory response of cultured human IECs. Among various endogenous secretagogues, we found that PGE_2_ had the lowest EC_50_ value with regard to the induction of swelling of the jejunal and colonic organoids. This PGE_2_-mediated swelling response was dependent on environmental Cl^−^ concentrations as well as on several channels and transporters as shown by a series of chemical inhibitor studies. The concomitant presence of various inflammatory cytokines with PGE_2_ failed to modulate the PGE_2_-mediated organoid swelling response. Therefore, the present study features PGE_2_ as a direct and robust mediator of anion/fluid secretion by IECs in the human intestine.

The intestinal mucosa plays an indispensable role in the homeostasis of anion and body fluid maintenance. The balance between fluid absorption and fluid secretion is finely regulated by various endogenous factors to maintain a proper inner-body fluid volume^1^. Studies have suggested that a gradient of these factors exists along the cephalocaudal axis and along the crypt-villus axis^2^,^3^.

Intestinal epithelial cells (IECs) directly regulate the absorption and release of fluids through the transport of anions in a basal-to-apical movement as well as an apical-to-basal movement. These functions of IECs are executed at the molecular level by the coordinated ion transport via membrane-bound transporters and channels at both the apical and basolateral membranes.

Fluid secretion by IECs is mainly regulated through the secretion of the Cl^−^ ion. The major transporter for Cl^−^ secretion on the apical surface of IECs is the cystic fibrosis transmembrane conductance regulator (CFTR)^4^. CFTR is activated through an increase of intracellular cAMP and thereby functions as a Cl^−^ channel as well as an HCO_3_^−^ channel^5^,^6^. Ca^2+^-dependent Cl^−^ channels (CaCCs) are another group of channels on the apical surface that can secrete Cl^− ^7. On the basolateral membrane, Na-K-2Cl cotransporter 1 (NKCC1) contributes to the uptake of Cl^−^, and the Na^+^/K^+^ ATPase and K^+^ channels, such as potassium voltage-gated channel subfamily Q member 1 (KCNQ1) and potassium calcium-activated channel subfamily N member 4 (KCNN4), support the function of NKCC1 by producing an electrochemical gradient across the luminal membrane^8^. Forced activation of this Cl^−^ secretion system by external stimuli, such as by the cholera toxin, leads to the excess secretion of fluids and manifests as a secretory-type diarrhoea^9^.

Various endogenous secretagogues are implicated in the regulation of Cl^−^ and fluid secretion by IECs. Neurotransmitters, such as acetylcholine (ACh) and vasoactive intestinal peptide (VIP), induce Cl^−^ secretion through muscarinic receptors and VIP receptors, respectively^10^,^11^. Serotonin and histamine are secreted by mast cells and promote Cl^−^ secretion by IECs through either a direct or indirect effect^12^,^13^. Additionally, the production of bradykinin is upregulated in the inflammatory environment and thereby promotes Cl^−^ secretion through B_2_ receptors expressed in IECs^14^.

Prostaglandin E_2_ (PGE2) is another endogenous secretagogue that may directly promote Cl^−^ secretion from IECs^15^. PGE_2_ is generated through the prostaglandin cascade and is mainly produced by mesenchymal cells in the intestinal mucosa^16^. The function of PGE_2_ is highly pleiotropic, as it can regulate the secretion of anions^17^ and mucus^18^, GI motility^19^, and the survival and proliferation of IECs^20^,^21^. PGE_2_ may also contribute to the maintenance of the stem-like properties of IECs through the enhancement of Wnt signalling^22^,^23^. In addition, PGE_2_ is one of the inflammatory mediators upregulated in inflammatory bowel disease (IBD)^24^,^25^. In the inflammatory environment, PGE_2_ may directly exert its effect on IECs and contribute to symptoms such as diarrhoea in IBD patients^26^. However, it is unclear to what extent PGE_2_ affects Cl^−^ secretion by human IECs. As most of the previously related studies were based on either carcinoma-derived cell lines, such as T84 or HT29, or on biopsies, there is a lack of data based on truly benign purified human IECs.

A recent advance in culture techniques has made it possible to maintain benign IECs *in vitro* for a desired period^27^,^28^,^29^. Crypt cells can be maintained in a 3D-structure referred to as organoids, and those organoids have been shown to serve as a suitable model to evaluate various IEC-specific functions^30^,^31^. A recent study suggested that the addition of forskolin (FSK) can induce forced fluid influx towards the inner space of the organoids and result in forskolin-induced swelling (FIS)^32^. Such a response is mediated by the function of CFTR and thus has been suggested as a useful index to measure its function in cystic fibrosis patients^33^. This study also showed that the FIS response might be extended to analyse the anion/fluid secretion of IECs that are triggered by other external stimuli.

In this study, we used the FIS technique and modified it to efficiently quantify the response of a large number of samples. Using our analysis system, we tested the response of human intestinal organoids based on cell samples from IBD patients to various endogenous secretagogues, including PGE_2_. Among the tested candidates, PGE_2_ showed the lowest EC_50_ value both in the small intestinal and the colonic organoids of inflammatory bowel disease patients. The observed swelling response to PGE_2_ appeared to be dependent on the environmental Cl^−^ concentration as well as on the presence of membrane-bound transporters or channels, such as CFTR, NKCC1, KCNQ1 and KCNN4. A panel of inflammatory cytokines completely failed to modify such a PGE_2_-mediated response, thus collectively indicating that PGE_2_ functions as a direct and robust mediator of Cl^−^ secretion by human IECs in various mucosal environments.

## Results

### Establishment of a quantitative screening method to evaluate the swelling response of intestinal organoids

To establish a method by which we can efficiently quantify the swelling of subject organoids in response to a large variety of conditions, we employed a newly developed 3D-scanning system to measure the cross-section area of the organoids (Supplementary Fig. S1). Our 3D-scanning system consists of a light emitting diode (LED)-type white light device positioned above the culture dish and a charge-coupled device (CCD) camera positioned underneath the dish; this camera can acquire a full-scan image of a single plate at a resolution of 4800 dpi within 1 min. First, to test the swelling response of our human intestine-derived organoids, FIS was examined. Throughout the present study, we mainly used intestinal organoids that were established from the uninflamed mucosa of patients with Crohn’s disease (CD). These organoids were maintained under stem/progenitor cell-enriched culture conditions as an *in vitro* model of crypt cells. The epithelial cell identity and stem/progenitor cell properties of the collected samples from the patients were confirmed by immunostaining for E-cadherin, pan-cytokeratin, Musashi-1^34^ and Ki-67 (Supplementary Fig. S2).

The shape of the organoids was either multilobular or spheroid depending on when the analysis was performed after the last passage; organoids analysed within 2 days post-passage predominantly presented with a spheroidal shape, while those analysed over 7 days post-passage predominantly showed a multilobular shape. These types of shape changes during the routine culture time course were repetitive and do not represent the variability among independent organoids at the same time points. Spheroidal organoids were consistently used for the following analyses using the 3D-scanner system. However, multilobular organoids were used in some of the pulse-chase experiments, as the dynamic change in shape was more demonstrative and clearly confirmed.

**Figure 1:**
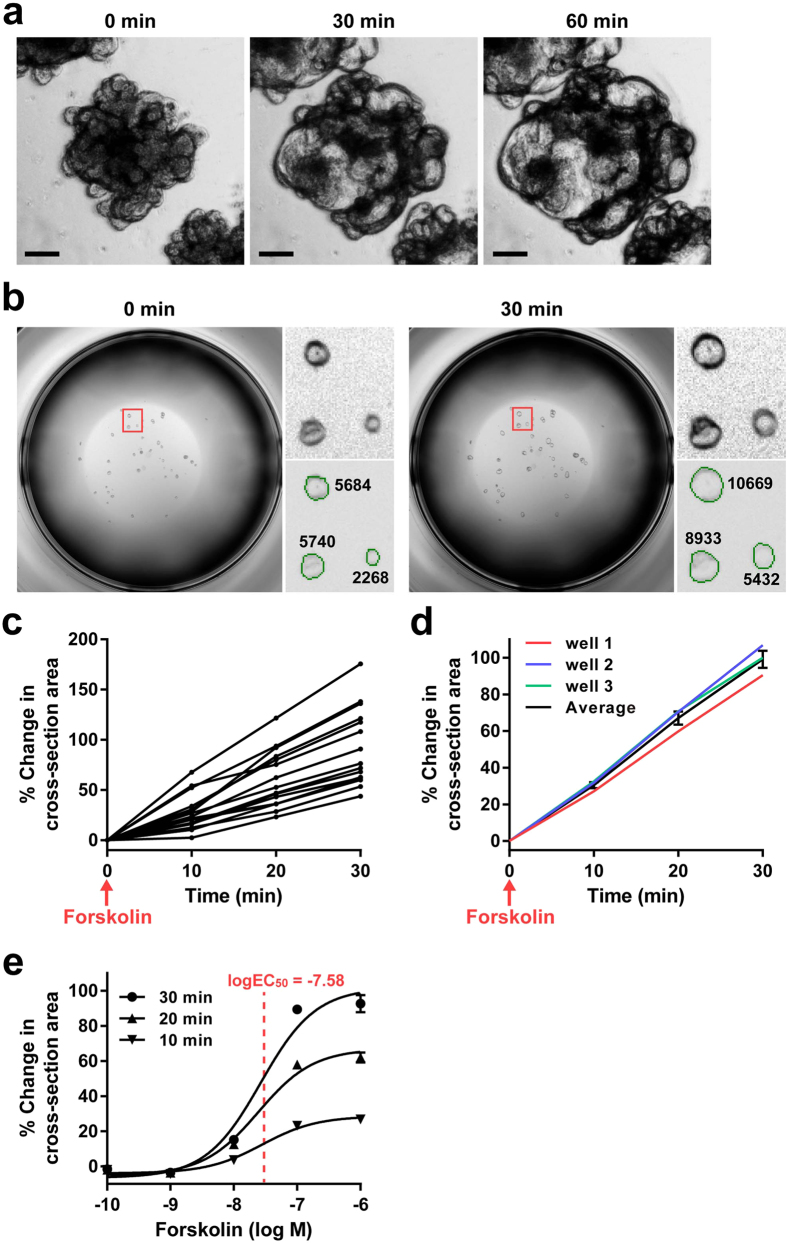
Establishment of a quantitative screening method to evaluate the swelling response of intestinal organoids. Human jejunal organoids were stimulated by forskolin to induce forskolin-induced swelling (FIS). (**a**) Phase-contrast view of the intestinal organoids at 10 days after passage shows continuous swelling in response to forskolin addition (10^−5^ M) for up to 60 min. Images were acquired by the EVOS-FL microscope. (**b**) Cross-section recognition and area measurement from the images acquired by the 3D-scanner. The image of a single well was acquired by the auto-focus (AF) mode of the scanner, and the cross-section area of each organoid was automatically cropped (green line) by optimizing the image recognition threshold. The corresponding area was measured both before and 30 minutes after forskolin addition (10^−6^ M). The magnified view of the area designated by the red square in the left panel is shown in the right panels. Numbers shown in the right lower panel indicate the measured area in μm^2^. (**c**) Monitoring of FIS (10^−6^ M) for up to 30 min by the 3D-scanning system illustrates the time-dependent swelling response at the single organoid level. (**d**) Comparison of the average swelling response induced by forskolin (10^−6^ M) among 3 individual wells using the % change in cross-section area derived from the total cross-section area of organoids per well. An average of the data acquired from the 3 independent wells is shown in the black line (mean ± SEM). (**e**) The dose-response curve of FIS was acquired by using a human jejunal organoid. It shows a sigmoid-shaped curve, and the logEC_50_ was determined as −7.58. All results are representative of at least three independent experiments.

Consistent with the former reports^32^,^35^, upon the addition of FSK, small intestinal organoids showed a rapid swelling response that continued for at least 60 min (Fig. 1a and Supplementary Movie S1). Further examination for up to 240 min revealed that organoids invariably show a continuous linear response up to 30 min after FSK stimulation (Supplementary Fig. S3). However, at time points longer than 30 min, the response curve showed a highly heterogeneous pattern among the organoids. Some of the organoids presented with a plateau pattern, suggesting that the response reached equilibrium. Other organoids showed a decrease in the response curve, indicating a collapse of the organoids (Supplementary Movie S2). From these preliminary data, we determined that measurements up to 30 min would be appropriate to examine the anion/fluid transport response of the organoids.

Using FIS as a positive control, we next tested if such a swelling response could be quantified by our 3D-scanning system. To optimize the quantification efficiency and accuracy, organoids were subjected to analysis one day after the last passage, as those organoids generally held their cystic shape at this time point. From here, the threshold to recognize the cross-section border of each organoid was optimized (Supplementary Table S1). Then, we confirmed that the optimized threshold setting accurately aligned with the cross-section border of each organoid (Fig. 1b), which would thereby precisely measure the cross-section area. By scanning the organoids before and after FSK addition, we calculated the increase in cross-section area of each organoid (% change in cross-section area as defined in the Material and Methods section) and reported this value as an index of the swelling response. Using the present system, we confirmed that the time-dependent swelling response can be quantitatively monitored at the single organoid level (Fig. 1c). Following the methods used by Dekkers *et al.*^32^, we also compared the response of individual wells to evaluate the inter-well difference of FIS (Fig. 1d). The results showed that the % change in cross-section area derived from the total cross-section area of organoids in a single well is highly conserved among the individual wells that were examined under the same conditions.

By running the present system, we could quantify the swelling response at a maximum output of 384 wells per single scan (Supplementary Fig. S1). This makes it possible for us to use the present system for drug response screening as well as determine the dose-response curve of a limited number of test reagents. Thus we tested whether the system could determine the dose-response curve of FSK. Using a human jejunum-derived organoid, we found that the dose-response curve for FIS appears as a standard sigmoid shaped curve, and the log EC_50_ value was calculated as −7.58 (Fig. 1e).

### Series of candidate endogenous mediators of anion/fluid secretion promotes the swelling of human jejunal organoids

**Figure 2:**
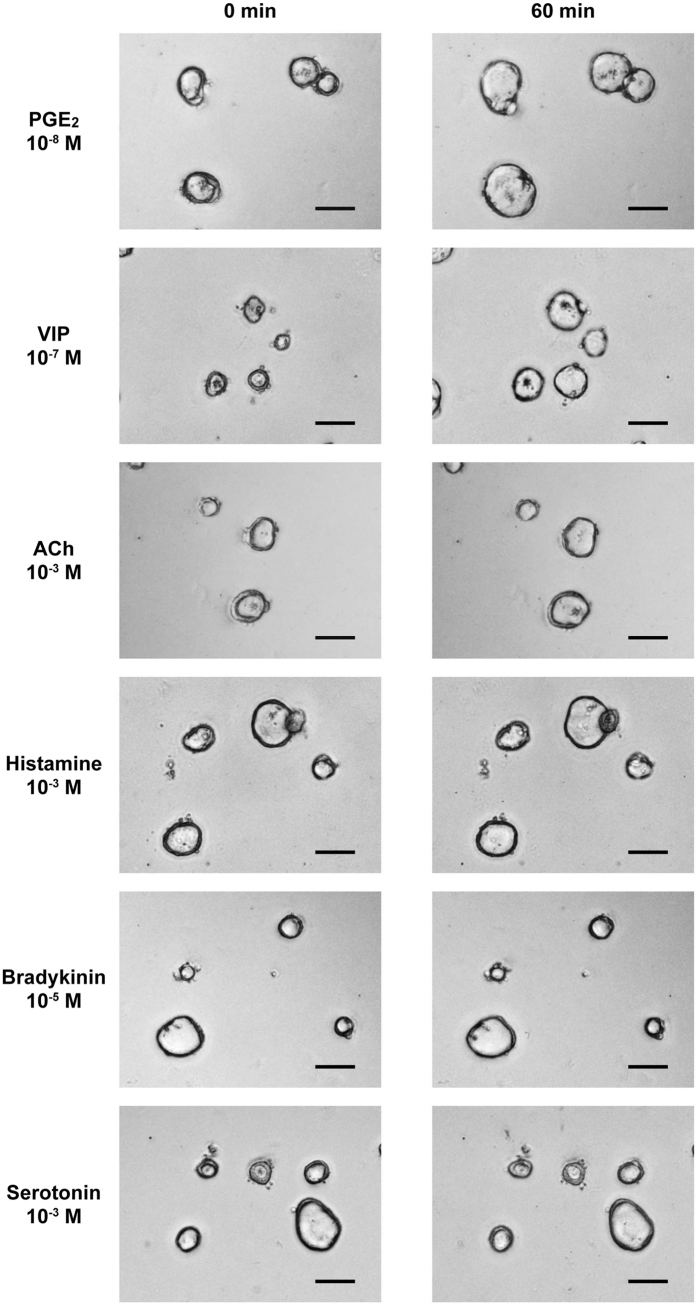
Series of candidate endogenous mediators of anion/fluid secretion promotes swelling of human small intestinal organoids. The induction of human jejunal organoid swelling by 6 different endogenous mediators of anion/fluid secretion was examined. Phase-contrast images acquired by the BZ-X700 microscope both before and 60 min after the induction are shown. Scale bar represents 100 μm. For the induction, PGE_2_ (10^−8^ M), VIP (10^−7^ M), ACh (10^−3^ M), histamine (10^−3^ M), bradykinin (10^−5^ M) or serotonin (10^−3^ M) was added to the culture medium.

To examine the direct effect of various endogenous mediators that may induce anion/fluid secretion from IECs, we tested 6 endogenous mediators to determine if they have the potential to induce the swelling response of organoids equivalent to that of FIS. Our time-lapse imaging showed marked differences among the mediators (Supplementary Movies S3–S8). The two mediators that function through Gs-coupled receptors, PGE_2_ and VIP, showed a rapid and continuous response that ended as a large overall increase in the cross-section area of the organoids at 60 min after induction (Fig. 2). In contrast, the mediators that function through Gq-coupled receptors, ACh and histamine, showed a slow and limited response, and the increase in the cross-section area of the organoids appeared to be smaller compared to that of either PGE_2_ or VIP. The addition of bradykinin or serotonin had no clear effect on the induction of organoid swelling.

**Figure 3:**
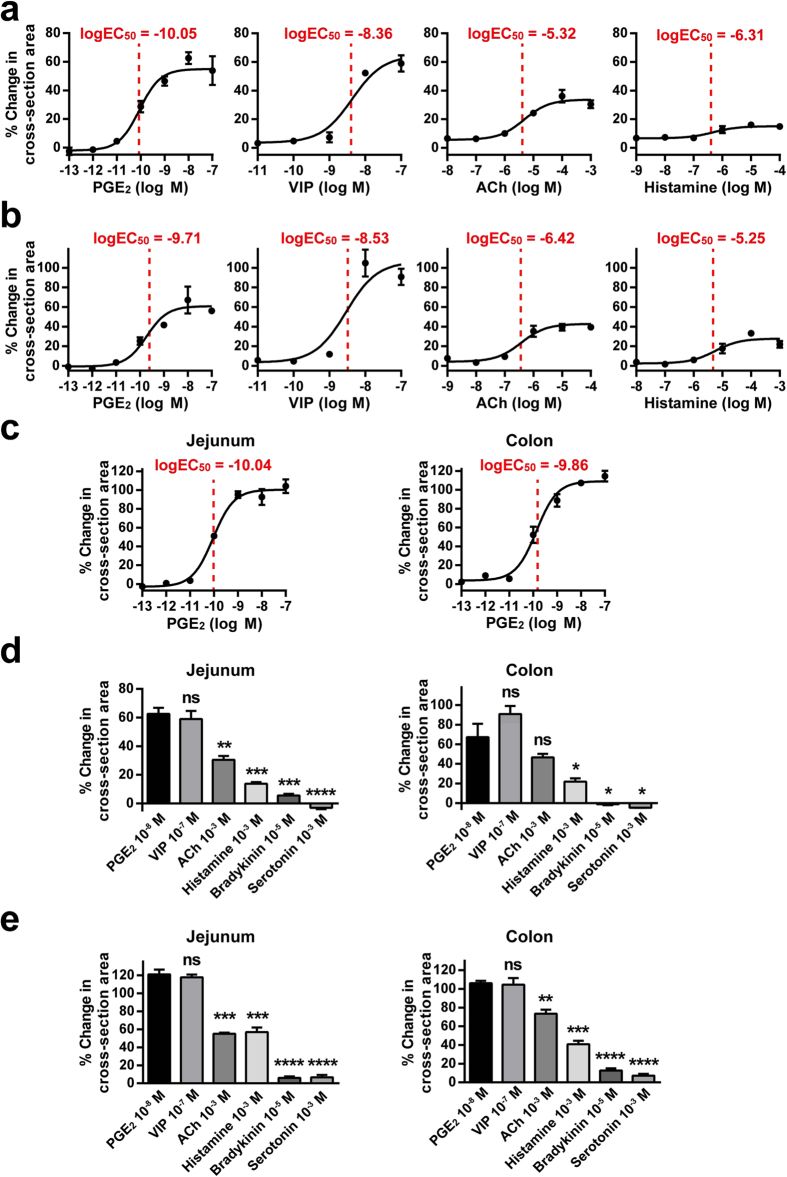
PGE_2_ functions as a key mediator of anion/fluid secretion in both the jejunum and the colon of the human gastrointestinal tract. Quantitative evaluation of the swelling response induced by PGE_2_, VIP, ACh and histamine was performed by the 3D-scanning system to acquire the dose-response curve of each reagent. (**a**) The dose-response curves acquired from human jejunum-derived organoids. (**b**) The dose-response curves acquired from human colonic organoids derived from the uninflamed mucosa of an ulcerative colitis (UC) patient. The organoids were established from a surgical specimen comprising several colonic segments. (**c**) The dose-response curves acquired from human jejunal and rectal organoids derived from the healthy mucosa of non-IBD patients. (**d**) The maximum response index values of the organoids used in (**a**,**b**) is compared among the tested mediators. (**e**) The maximum response index values of the organoids used in (**c**) is compared among the tested mediators. Data are shown as the mean ± SEM of three independent wells. *indicates P < 0.05, **indicates P < 0.005, ***indicates P < 0.0005, ****indicates P < 0.0001 as determined by two-sided Student’s t-test compared to the data of PGE_2_. ns indicates not significant. All results are representative of at least three independent experiments.

To confirm the data acquired by time-lapse imaging, we next tried to examine the difference in the dose-response curves of mediator-induced organoids. First, we used a human jejunal organoid to evaluate the direct response of these mediators. The results showed a clear sigmoid-shaped response curve for PGE_2_ and VIP, while a much lower response profile was observed for ACh and histamine (Fig. 3a). This type of response pattern was also generally conserved in the human colonic organoids derived from the uninflamed mucosa of a patient with ulcerative colitis (UC) (Fig. 3b). Thus, these results showed that PGE_2_ could induce the swelling of jejunal and colonic organoids at the lowest log EC_50_ value of the mediators tested. In addition, the dose-response curves and log EC_50_ values of PGE_2_ were reproduced and confirmed in another line of jejunal and colonic organoids established from a single CD patient (Supplementary Fig. S4) as well as in organoids established from the intact ileal mucosa of a UC patient (Supplementary Fig. S5), suggesting that our present data may be common among the uninflamed mucosa of IBD patients. Importantly, organoids that were established from the healthy mucosa of non-IBD patients also showed an equivalent response to PGE_2_ (Fig. 3c). Of note, the EC_50_ value identified for PGE_2_ in the jejunal organoids appeared to be 100-fold lower than that of FSK (Fig. 1e). In both the jejunum and the colon, the response to PGE_2_ also showed the highest values (Fig. 3d,e). Therefore, among the various endogenous mediators of anion/fluid secretion, PGE_2_ functions as one of the key inducers of anion/fluid secretion due to its direct effect on IECs.

Our organoids consistently and clearly expressed the PGE_2_ receptor subtypes EP_1_, EP_2_ and EP_4_ (Supplementary Fig. S6). We further examined if other types of prostaglandins, such as PGD_2_, may have the potential to induce the swelling of human small intestinal organoids (Supplementary Fig. S6). The results showed that PGA_2_ and PGE_1_ may have an equivalent potential to induce the swelling of human organoids. However, previous reports have indicated that PGE_2_ has the highest potency of intestinal fluid secretion among eicosanoids^36^. Additionally, the local production of PGE_2_ but neither the production of PGA_2_ nor PGE_1_ is clearly upregulated in the inflamed mucosa of IBD patients^24^,^36^,^37^, suggesting that PGE_2_ may be the dominant eicosanoid mediator of anion/fluid secretion in these patients.

### PGE_2_-induced organoid swelling is dependent on Cl^−^

**Figure 4:**
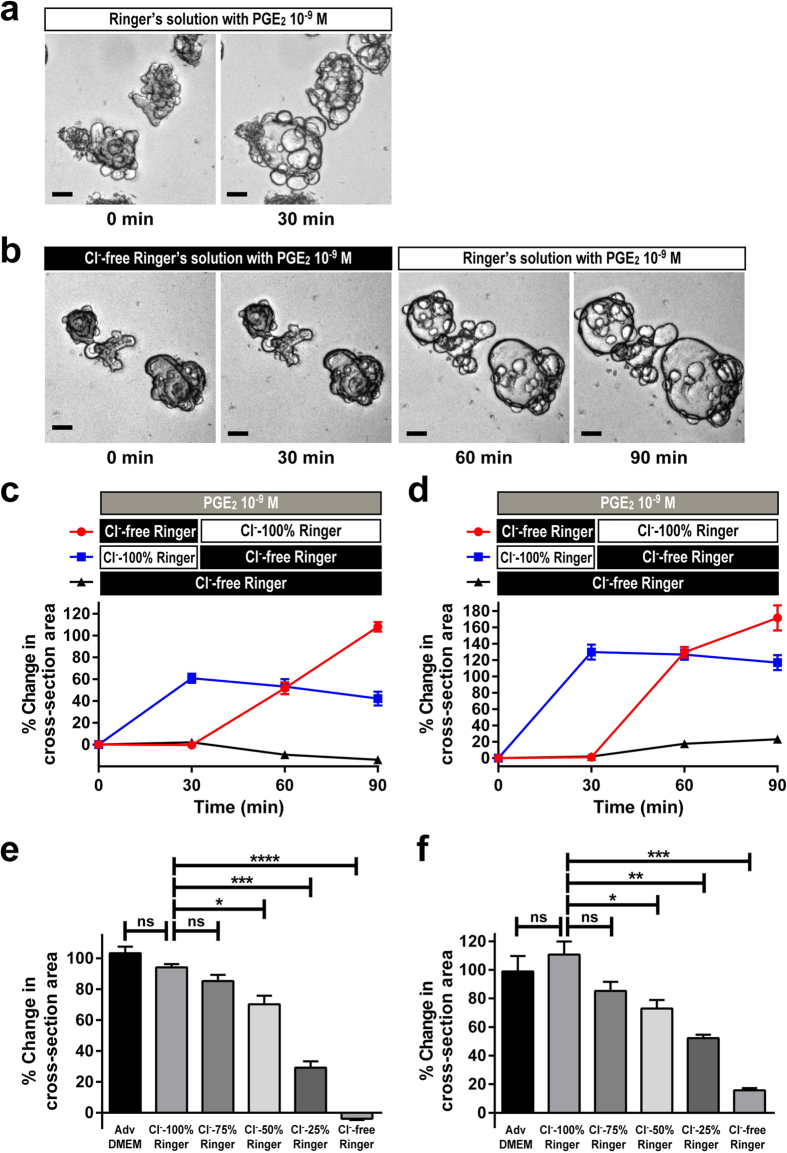
PGE_2_-induced organoid swelling is dependent on Cl^−^. The PGE_2_-induced swelling of jejunal organoids was tested under normal, Cl^−^ reduced and Cl^−^ depleted buffer conditions. (**a**) Phase contrast view of the PGE_2_-induced swelling in Ringer’s solution supplemented with standard Cl^−^ level (126.8 mEq). PGE_2_ (10^−9^ M) was added to jejunal organoids for 30 min at 7 days after passage. Note that the swelling response is completely maintained. Scale bar represents 100 μm. (**b**) Phase contrast view of the PGE_2_-induced swelling in Cl^−^-free Ringer’s solution. PGE_2_ (10^−9^ M) was added to jejunal organoids for 30 min at 7 days after passage. Note that the swelling response is completely abolished by Cl^−^ depletion, but can be restored by the following addition of buffer supplemented with Cl^−^ (126.8 mEq). Scale bar represents 100 μm. (**c**,**d**) Time course experiment lasting up to 90 min showing the swelling response to PGE_2_ under normal or Cl^−^-free Ringer’s solution. Jejunal organoids established from the uninflamed region of a CD patient (**c**) and those established from the healthy mucosa of a non-IBD patient (**d**) were subjected to the PGE_2_-induced swelling (10^−9^ M) under normal or Cl^−^-free Ringer’s solution, and the response was quantified by the 3D-scanning system. (**e,f**) Quantification of the PGE_2_-induced swelling under different Cl^−^ concentrations. Jejunal organoids established from the uninflamed region of a CD patient (**e**) and those established from the healthy mucosa of a non-IBD patient (**f**) were subjected to the PGE_2_-induced swelling (10^−9^ M) under different Cl^−^ concentrations, and the response was quantified by the 3D-scanning system. Data are shown as the mean ± SEM of three independent wells. *indicates P < 0.05, ***indicates P < 0.0005, ****indicates P < 0.0001 as determined by two-sided Student’s t-test compared to the data of control (Adv DMEM; Advanced DMEM) or Cl^−^ 100% Ringer. ns indicates not significant. All results are representative of at least three independent experiments.

Studies have shown that fluid secretion by IECs is mediated by the secretion of anions such as Cl^− ^4. To identify if the previously observed response induced by PGE_2_ is mediated by the secretion of Cl^−^, we examined the response in Cl^−^-free Ringer’s solution. In Ringer’s solution supplemented with 126.8 mEq of Cl^−^, the response of jejunal organoids to PGE_2_ was clearly conserved (Fig. 4a). However, when those organoids were placed in Cl^−^-free Ringer’s solution, the PGE_2_-induced swelling response was completely eliminated (Fig. 4b). The abolishment was not due to the loss of cell viability under the Cl^−^-free environment, as the response was completely restored by adding buffer supplemented with Cl^−^ (Fig. 4b). A time-course experiment lasting up to 90 min using jejunal organoids established from either the uninflamed mucosa of a CD patient (Fig. 4c) or the healthy mucosa of a non-IBD patient (Fig. 4d) further showed that a clear swelling response could be neither induced nor maintained in organoids that were placed in a Cl^−^-free environment, but the organoids regained their swelling response once they were placed back into Cl^−^-supplemented Ringer’s solution. Additionally, the dependence on the Cl^−^ concentration was further confirmed by quantification of the response at different levels of Cl^−^ using organoids of both origins (Fig. 4e,f). Thus, these results support the idea that the PGE_2_-induced organoid swelling is dependent on the induction of Cl^−^ secretion from organoid-based IECs.

Previous reports have indicated that HCO_3_^−^ may also contribute to the secretory process of IECs^35^. Thus, we further tested whether HCO_3_^−^ may play a role in the present swelling response by adding HCO_3_^−^ to Cl^−^- and HCO_3_^−^-free Ringer’s solution (Supplementary Fig. S7). Consistent with the previous experiment, depletion of both Cl^−^ and HCO_3_^−^ completely abrogated the swelling response. Supplementation of Cl^−^ almost completely restored the swelling response, whereas supplementation of HCO_3_^−^ partly restored the swelling response to a minimal extent. Thus we conclude again that the secretory response is predominantly dependent on Cl^−^ secretion, accompanied by a minor contribution of HCO_3_^−^ secretion.

### PGE_2_-induced organoid swelling is sensitive to inhibitors targeted to CFTR, NKCC1, KCNQ1 and KCNN4

**Figure 5:**
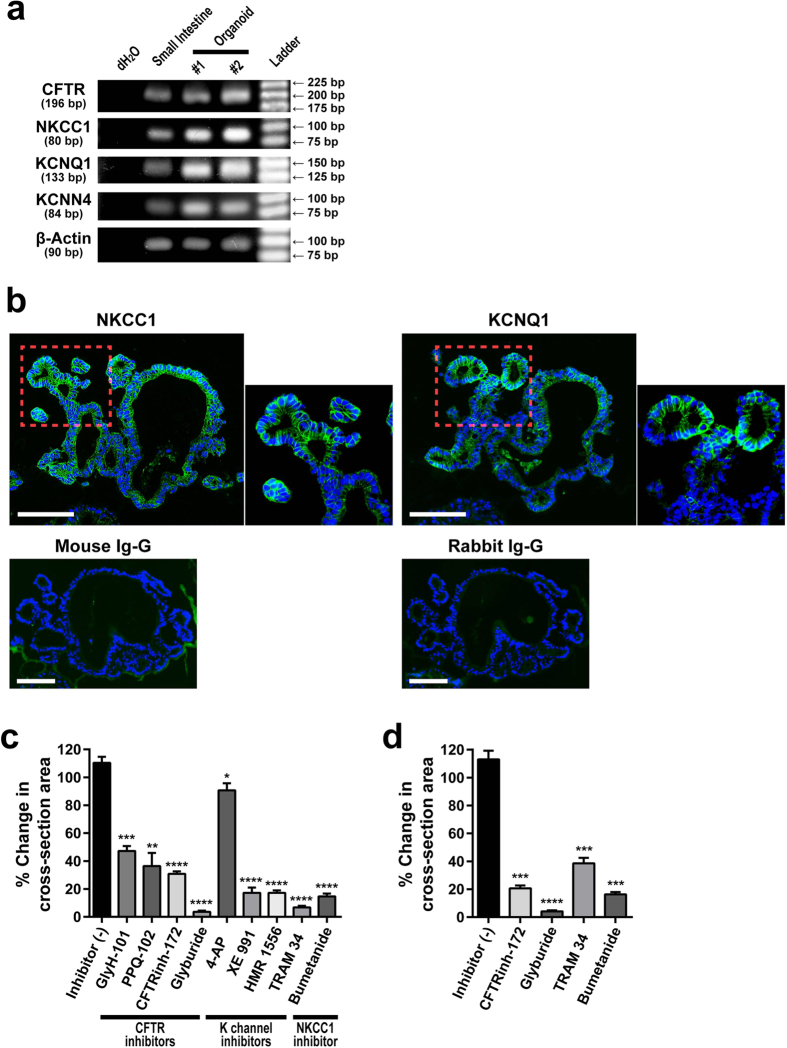
PGE_2_-induced organoid swelling is sensitive to inhibitors targeted to CFTR, NKCC1, KCNQ1 and KCNN4. (**a**) mRNA of membrane-bound ion channels and transporters are expressed in cultured human small intestinal organoids. Semi-quantitative RT-PCR analysis shows expression of CFTR, NKCC1, KCNQ1 and KCNN4 in small intestine-derived organoids. Results acquired from the whole small intestinal tissue sample are shown as a reference. dH_2_O served as a negative control. The number of bp indicates the length of the targeted amplification region for each gene. (**b**) Immunostaining of NKCC1 and KCNQ1 shows their expression at the basolateral membrane of cultured small intestinal organoids. Organoids were fixed and subjected to immunostaining using primary antibodies specific for NKCC1 or KCNQ1. The expression was visualized by FITC-labeled Tyramide (green). The stainings using non-immunized mouse IgG and rabbit IgG served as negative controls. Scale bar represents 100 μm. (**c,d**) Inhibitory effects of various compounds on the PGE_2_-induced swelling of jejunal organoids that were established from the uninflamed region of a CD patient (**c**) and from the healthy mucosa of a non-IBD patient (**d**). Inhibitors were added 1 hour prior to PGE_2_-induction at the following concentration: GlyH-101 (50 μM), PPQ-102 (10 μM), CFTR Inhibitor-172 (CFTRinh-172) (50 μM), glyburide (500 μM), 4-AP (1 mM), XE 991 (10 μM), HMR 1556 (10 μM), TRAM 34 (10 μM) and bumetanide (100 μM). PGE_2_-induced swelling was quantified 30 min after the PGE_2_-induction (10^−9^ M). Data are shown as the mean ± SEM of three independent wells. *indicates P < 0.05, **indicates P < 0.005, ***indicates P < 0.0005, ****indicates P < 0.0001 as determined by two-sided Student’s t-test compared to the data of Inhibitor (−). All results are representative of at least three independent experiments.

Various transporters and channels that are expressed on the apical and basolateral membranes of IECs constitute the molecular mechanism that mediates anion/fluid secretion by the intestinal epithelium. Therefore, we examined if these molecules are expressed and functional in our organoids. RT-PCR analysis showed that mRNA expression of CFTR, NKCC1, KCNQ1 and KCNN4 are clearly detected in the small intestinal organoids (Fig. 5a). Within these organoids, the expression of NKCC1 and KCNQ1 was confirmed at the protein level, as the immunostaining of those molecules showed clear localization at the basolateral membrane (Fig. 5b).

We next examined whether these membrane-bound channels or transporters contributed to PGE_2_-induced organoid swelling. A series of inhibitors targeted to CFTR showed a clear inhibitory effect on the PGE_2_-induced swelling of jejunal organoids (Fig. 5c). Although the inhibitory effect varied among the compounds, addition of glyburide (500 μM) showed a marked abolishment of this response, suggesting that the PGE_2_-induced Cl^−^ secretion on the apical side might be highly dependent on CFTR. In addition, we found that inhibitors specific for NKCC1, KCNQ1 or KCNN4 can also partially reduce PGE_2_-induced swelling (Fig. 5c), indicating that Cl^−^ uptake on the basolateral side requires cooperative function of these channels and transporters. A similar effect of the inhibitors targeted to CFTR, the K^+^ channel or NKCC1 was confirmed not only in the jejunal organoids that were established from the uninflamed mucosa of an IBD patient but also in organoids that were established from the healthy mucosa of a non-IBD patient (Fig. 5d).

Studies have suggested that growth factors such as epidermal growth factor (EGF) may confound the properties of the IECs by dysregulating the expression of anion channels or transporters^38^. However, depletion of EGF for up to 24 hours did not change the response of PGE_2_-induced swelling (Supplementary Fig. S8). Thus, our results so far indicate a limited influence of growth factors such as EGF on PGE_2_-induced swelling.

### PGE_2_-induced organoid swelling cannot be modulated by inflammatory signals

**Figure 6:**
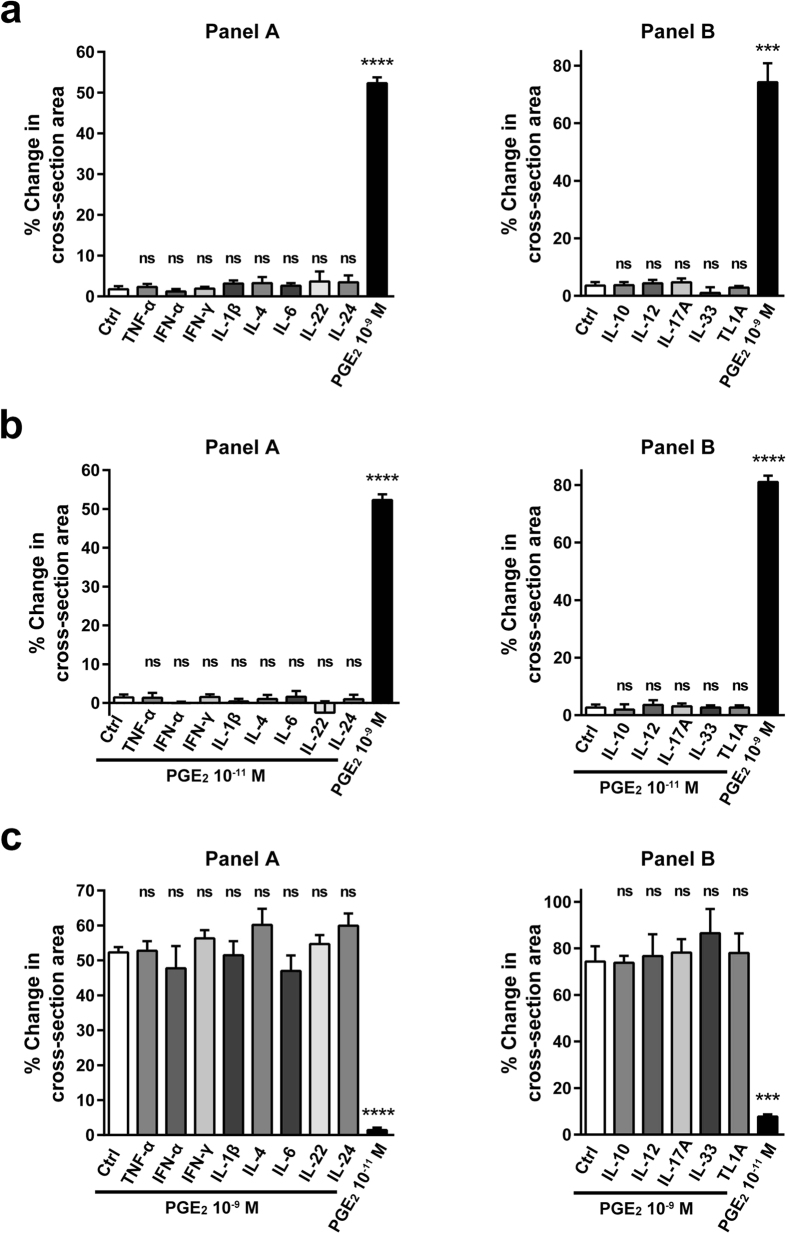
PGE_2_-induced organoid swelling cannot be modulated by inflammatory signals. PGE_2_-induced swelling was quantified under the concomitant presence of various pro-inflammatory cytokines. The concentration of each cytokine is as follows: TNF-α (100 ng/ml), IFN-α (50 ng/ml), IFN-γ (10 ng/ml), IL-1β (20 ng/ml), IL-4 (10 ng/ml), IL-6 (50 ng/ml), IL-22 (20 ng/ml), IL-24 (100 ng/ml), IL-10 (10 ng/ml), IL-12 (20 ng/ml), IL-17A (50 g/l), IL-33 (10 ng/ml) and TL1A (100 ng/ml). Panel A and Panel B represent a series of data acquired by the scanning of a 96-well plate. (**a**) A panel of cytokines were incubated with the jejunal organoids for 30 min, and examined for their ability to induce organoid swelling without PGE_2_. High-dose PGE_2_ (10^−9^ M) served as a positive control. (**b,c**) The same panel of cytokines as shown in (**a**) was pre-incubated with the jejunal organoids for 30 min, and then swelling was induced by low-dose PGE_2_ (10^−11^ M) (**b**) or high-dose PGE_2_ (10^−9^ M) (**c**). Data are shown as the mean ± SEM of three independent wells. ***indicates P < 0.0005, ****indicates P < 0.0001 as determined by two-sided Student’s t-test compared to the data of control. ns indicates not significant. All results are representative of at least three independent experiments.

PGE_2_ is a mediator whose expression is upregulated in different IBDs, such as UC or CD^24^,^39^,^40^. In such an environment, the response of IECs could be modified by the concomitant presence of various pro-inflammatory cytokines^41^. Therefore, we examined whether the present PGE_2_-induced organoid swelling could be modified by the presence of inflammatory cytokines. Short-term pretreatment (30 min) of the jejunal organoids with cytokines such as TNF-α, IL-1β, IL-6, IL-22 or IFN-γ alone failed to induce a swelling response (Fig. 6a). When PGE_2_-induced swelling was tested following the cytokine pretreatment, the response level of both low-dose PGE_2_ (10^−11^ M) and high-dose PGE_2_ (10^−9^ M) remained completely unchanged (Fig. 6b,c). We further examined the effect of up to 13 major cytokines that are relevant to IBD and found that all the tested cytokines exerted absolutely no effect on PGE_2_-induced swelling. These results indicate that PGE_2_ may act as a robust and direct mediator of anion/fluid secretion from IECs, which cannot be affected by other pro-inflammatory cytokines. Additionally, the results raise the possibility that the local levels of PGE_2_ may constitute one of the components in the pathophysiology of IBD-associated diarrhoea^42^,^43^.

## Discussion

In the present study, we established a modified method to quantitatively evaluate the swelling of human intestinal organoids. Our method employed a 3D-scanner that can quantify the swelling response of a large number of samples simultaneously in a 96-well plate format. The current method did not use fluorescent staining to detect the inner rim of the organoids but instead used an auto-focused phase-contrast image of a single well. Using the ratio of cross-section area as an index of swelling, we acquired experimental results of FIS treatment that are consistent with previous reports^32^,^35^,^44^, using human intestinal organoids. In the experiments analysing FIS, all the organoids responded without exception and resulted in a complete response of the subject organoids (Fig. 1c). However, if we included other replicates of the experiment, 1 out of 63 subject organoids failed to respond to forskolin (1.53% of total organoids). Such a low rate of nonresponsive organoids compared to that from a previous report^32^, may be due to differences in the culture conditions, passage methods or background characteristics of the source from which the organoids were derived.

The present method may be further applied to test the swelling response of a wide variety of compounds or natural ligands and to efficiently evaluate their potential to induce or otherwise inhibit the anion/fluid secretion of IECs. Among the examined endogenous secretagogues, VIP and PGE_2_ showed a clear and extended response, whereas ACh, histamine, bradykinin and serotonin showed a somewhat weak and unsustained response. This profound swelling response after PGE_2_ addition was generally conserved between organoids established from the non-inflamed region of IBD patients and organoids established from the healthy mucosa of non-IBD patients. The differential response among the examined secretagogues may arise from the difference in the intracellular signalling of the corresponding receptors. The receptors for VIP and PGE_2_ are Gs-coupled receptors both mediate cAMP intracellular signalling, while the receptors for ACh, histamine, bradykinin or serotonin are Gq-coupled receptors, which mediate Ca^2+^-based intracellular signalling^1^. Gq-coupled receptors undergo tachyphylaxis and thus cannot sustain their response under a continuous ligand presence *in vitro*. Another possibility is the difference in the expression levels of the corresponding receptors. Regarding the response to PGE_2_, EP_2_ and EP_4_ may be the dominant receptors in IECs^45^,^46^. Clear expression of both EP_2_ and EP_4_ was consistently found in our organoids (Supplementary Fig. S6). However, further study using a set of EP agonists and antagonists may reveal the receptor that is responsible for the observed swelling response. Additionally, our finding that PGE_2_-induced swelling can be completely nullified by removing all Cl^−^ from the buffer indicates the great dependency of this activity on Cl^−^ uptake and secretion in the basal-to-apical direction.

The attempts of our study to identify the transporters and channels that are responsible for the PGE_2_-induced swelling showed that CFTR might be the dominant transporter on the apical membrane. Among the CFTR inhibitors, glyburide showed the most significant inhibitory effect on PGE_2_-induced swelling (Fig. 5c,d). Glyburide is a compound classified as a second-generation sulfonylurea antidiabetic drug. It can act as an inhibitor of ATP-sensitive potassium channels (Kir6.1 and Kir6.2, KATP) in pancreatic β-cells in addition to its activity on CFTR according to the IUPHAR database. Although the expression of neither Kir6.1 nor Kir6.2 has been identified in human IECs, it remains possible that glyburide inhibits swelling of the organoids through the inhibition of ATP-sensitive potassium channels such as Kir6.1 or Kit6.1 in addition to its activity on CFTR.

In addition, the inhibitor study showed that NKCC1, KCNQ1 and KCNN4 might contribute to the response on the basolateral membrane. Both NKCC1 and KCNQ1 are exclusively expressed in crypt-resident cells but not in those cells residing at the villi^35^,^47^. Thus our results suggest that cooperation of these transporters is induced specifically in crypt IECs upon local secretion of PGE_2_. The possible involvement of other channels, such as ClC-2 or CaCCs^48^ remains to be elucidated. However, the existence and the role of CaCCs in the intestinal epithelia still remains controversial^49^, and these channels may have a more dominant role in intestinal motility^50^. Additionally, the residual secretion observed in each inhibitor study suggests the possibility that the partial compensation of off-target transporters/channels or the reciprocal activation of an as of yet unknown transporter/channel system is induced in IECs. Although the inhibitors were tested at a concentration far above their IC_50_, a possibility remains that the residual secretion is due to their partial effect on the target channel or transporter.

Surprisingly, the response to PGE_2_ was robust and minimally influenced by short-term treatments with inflammatory cytokines. With regard to the pathophysiology of inflammatory diarrhoea, transport of Cl^−^ may represent a partial component, as other factors such as disruption of the mucosal integrity or an increase of exudates arising from the damaged mucosa may also constitute the major components of inflammatory diarrhoea. However, expression of PGE_2_ is upregulated in the inflammatory lesions of UC and CD patients, where other inflammatory cytokines are also abundant^51^. Thus our present results suggest the possibility that the local PGE_2_ concentration may determine the local secretory fluid response of IECs without the involvement of other inflammatory cytokines and thereby partially contribute to the pathophysiology of inflammatory diarrhoea. Consistently, none of the cytokines tested in the present study was able to induce organoid swelling. Further studies using organoids established from the diseased mucosa of IBD patients may provide insights to the role of PGE_2_ in the pathophysiology of IBD.

The current organoid system may have some superior points compared to other existing systems used for transport measurement^52^. For example, biopsies mounted in Ussing chambers could be used to perform experiments that mimic physiological conditions. Such a system may be better suited for electrophysiological studies to discriminate the contribution of each anion and to evaluate the overall effect of the mucosa including cells other than IECs. Additionally, these chambers can be easily applied to analyse the possible differences in responses at the apical or basolateral lumen. However, it may be better to use the organoid system when one wants to examine the direct effect of the mediators to epithelial cells, and wish to employ the volume of water secretion as a readout of the response. Additionally, the use of organoids has the following advantages: (1) the cultures are viable for a longer period with proper maintenance, (2) they can be analysed under differentiated or undifferentiated culture conditions^35^, and (3) they can undergo semi-high throughput screening (as shown in the present study). Currently it is difficult to access the inner lumen of organoids, which makes manipulating the IECs from the apical lumen one of the major disadvantages of using the organoids.

Finally, we would like to emphasize the caveat of using the current organoid swelling model to evaluate fluid secretion by IECs. First, our present condition mainly reflects the response of crypt cells and minimally accounts for the contribution of villus cells. Second, the quantitative evaluation was carefully restricted to regularly shaped organoids, as the current assay estimates the influx fluid volume from the representative cross-section area. Third, the possibility of fluid leakage through a weakened or fragile section of the epithelial monolayer should be carefully considered, especially when an inhibitory effect is observed.

## Material and Methods

### Establishment and culture of human intestinal organoids

Human intestinal biopsy specimens were obtained from patients who underwent enteroscopic examination for the evaluation of diseases such as occult bleeding, irritable bowel syndrome, Crohn’s disease and ulcerative colitis. Two or three biopsies were taken from an endoscopically normal region. Surgical specimens of ulcerative colitis patients were also collected to establish organoids. The study was approved by the Ethics Committee of Tokyo Medical and Dental University and the Yokohama Municipal Citizens Hospital, and written informed consent was obtained from each patient. A total of 38 lines of small intestinal (non-IBD origin, n = 3; IBD origin, n = 20) or colonic organoids (non-IBD origin, n = 4; IBD origin, n = 11) were established from 27 patients. All the experiments were conducted in accordance with the approved guidelines. Throughout the present study, unless otherwise indicated, we mainly used intestinal organoids that were established from the uninflamed mucosa of patients with CD. Additionally, we never used organoids established from endoscopically active diseased mucosa of IBD patients.

Isolation of the crypts and the subsequent establishment of intestinal organoids were performed as previously described^28^,^53^. Briefly, crypts were collected by rigorously shaking biopsy specimens in 2.5 mM EDTA. Isolated crypts were embedded in 15 μl of Matrigel at a density of 20–30 crypts per well and placed in either a 24-well or 48-well culture dish. Those crypts were maintained in DMEM-based culture medium (Advanced-DMEM, Invitrogen, California, USA) supplemented with recombinant human R-spondin-1 (1 μg/ml, R&D Systems, Minneapolis, USA), recombinant human Wnt-3a (300 ng/ml, R&D Systems, Minneapolis, USA), recombinant human Noggin (100 ng/ml, R&D Systems, Minneapolis, USA), recombinant human EGF (50 ng/ml, PeproTech, USA), Y-27632 (10 μM, Sigma-Aldrich Japan, Tokyo, Japan), A83-01 (500 nM, Sigma-Aldrich Japan, Tokyo, Japan) and SB202190 (10 μM, Sigma-Aldrich Japan, Tokyo, Japan). These culture conditions maintained the organoids in an undifferentiated state, and thus allowed us to define the secretory function of human IECs using those organoids.

Cl^−^-free Ringer’s solution and HCO_3_^−^-supplemented Ringer’s solution were prepared as previously described^54^,^55^. The composition of each solution is shown in detail in Supplementary Table S2. The chemicals and reagents that were used in the present study were as follows: PGA_2_, PGD_2_, PGE_1_, PGE_2,_ PGF_2_α, PGI_2_ and bumetanide (Cayman Chemical, Michigan, USA); VIP, ACh, histamine, bradykinin and serotonin hydrochloride (Sigma-Aldrich Japan, Tokyo, Japan); glyburide, GlyH-101, CFTRinh172 and PPQ-102 (Santa Cruz, Texas, USA); XE 991 dihydrochloride, 4-AP, HMR 1556 and TRAM 34 (Tocris Bioscience, Bristol, UK); recombinant human IL-10, IL-12, IL-17, IL-33 and TL1A/TNFSF15 (R&D Systems, Minneapolis, USA); and recombinant human TNF-α, IFN-α, IFN-γ, IL-1β, IL-4, IL-6, IL-22 and IL-24 (PeproTech, Rocky Hill, USA). The phase-contrast images of cultured cells were collected by using a microscope (BZ-X700, KEYENCE, Tokyo, Japan). The microscope is an inverted type microscope system dedicated to bright-field and fluorescence imaging and is equipped with a 3.7 W LED and 80 W metal halide lamp. Objective lenses from the Nikon CFI60 series were used for the present study (CFI Plan Apo λ 4x, NA 0.20; CFI Plan Apo λ 10x, NA 0.45; CFI Plan Apo λ 20x, NA 0.75; CFI Plan Apo λ 40x, NA 0.95).

### Quantification of organoid swelling

The quantification of organoid swelling was performed using a 3D-Scanner (Cell3 iMager, Screen Holdings, Kyoto, Japan). Organoids were seeded into a 96-well plate for the 3D-scanning with 2 μl of Matrigel and 100 μl of complete culture medium. On the next passage day, scanning was performed both before and 30 min after the addition of the reagent that was subjected for analysis. Upon scanning, an image was acquired by the auto-focus (AF) mode. The total cross-section area of the recognized organoids was automatically determined by setting the analysis parameters as summarized in Supplementary Table S1. Briefly, both the “Edge detection” mode and the “Include high light area” mode were the most critical settings for the present analysis (Supplementary Fig. S1). The index of organoid swelling acquired at the specified time in minutes (% change in cross-section area) was calculated by the following formula:
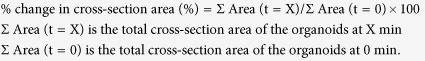


### Time-lapse imaging

Time-lapse imaging of the organoids was performed by using the EVOS-FL cell imaging system (Thermo Fisher Scientific, Waltham, MA, USA). Cells were maintained in a stage top incubator (Tokai Hit, Shizuoka, Japan) at 5% CO_2_ and 37 °C. Phase-contrast images were acquired at 1-min intervals for up to 60 min and were processed into a video file using the EVOS-FL system software (Thermo Fisher Scientific, Waltham, MA, USA).

### RT-PCR analysis

Semi-quantitative RT-PCR analysis was done as previously described^56^. The PCR reaction was run by using a Veriti thermal cycler system (Applied Biosystems, Waltham, MA, USA). The primer sequences for human β-actin have been previously described^56^. Primer sequences for other genes are as follows: EP_1_, 5-CTTCGGCCTCCACCTTCTTT-3 (sense) and 5-GCCACCAACACCAGCATTG-3 (antisense); EP_2_, 5-GCTCCTTGCCTTTCACGATTT-3 (sense) and 5-AGGATGGCAAAGACCCAAGG-3 (antisense); EP_3_, 5-CTTCGCATAACTGGGGCAAC-3 (sense) and 5-TCTCCGTGTGTGTCTTGCAG-3 (antisense); EP_4_, 5-CGCTCGTGGTGCGAGTATT-3 (sense) and 5-CCCGCCAATGCGGCAG-3 (antisense); CFTR, 5-CGCCCGAGAGACCATGC-3 (sense) and 5-CAGCTCTCTATCCCATTCTCTTT-3 (antisense); NKCC1, 5-ACACACAAAGTTGAGGAAGAGGA-3 (sense) and 5-GGCACAATAGGGCCTTTGGA-3 (antisense); KCNQ1, 5-CGTCTCCATCTACAGCACGC-3 (sense) and 5-CAGGACGATGAGGAAGACGG-3 (antisense); and KCNN4, 5-TGCACGATCAGCATTTCCAC-3 (sense) and 5-GTCGGTCATGAACAGCTGGA-3 (antisense). Semi-quantitative analysis was performed by the following numbers of PCR cycles: 34 cycles for EP_1_, 32 cycles for EP_2_, 34 cycles for EP_3_, 32 cycles for EP_4,_ 32 cycles for CFTR, 28 cycles for NKCC-1, 31 cycles for KCNQ-1, 28 cycles for KCNN-4 and 20 cycles for β-actin. The amplified products were separated by electrophoresis in a 3% agarose gel and visualized by ethidium bromide staining. Stained images were then acquired by using the ChemiDoc imaging system (Bio-Rad Laboratories, Hercules, CA, USA).

### Immunocytochemistry

Immunostaining of the organoids was performed as previously described^29^. The following primary antibodies were used in the present study: rabbit anti-human KCNQ1 (H-130) antibody (1:300, Santa Cruz, Texas, USA), mouse anti-human SLC12A2/NKCC1 (5H7) antibody (1:100, LSBio, Washington, USA), mouse anti-human E-cadherin (HECD-1) antibody (1:100, Takara, Tokyo, Japan), mouse anti-human pan-cytokeratin antibody (1:50, clone AE1/AE3, DAKO, Glostrup, Denmark), rat anti-human Musashi-1 antibody (1:1000, 14H1, eBioscience) and mouse anti-human Ki-67 antibody (1:50, DAKO, Glostrup, Denmark). The primary antibodies were visualized with either Alexa Fluor^®^ 488-conjugated secondary antibodies (Thermo Fisher Scientific, California, USA) or FITC-conjugated tyramides (Perkin Elmer, Waltham, USA). Signal amplification was required to detect NKCC1, KCNQ1 and Musashi-1. Images were collected by using an epifluorescent microscope (BZ-X700, KEYENCE, Tokyo, Japan).

### Statistics

Unless otherwise indicated, the data were confirmed by at least three independent experiments, each consisting of three replicates acquired from three independent culture wells. Data are shown as the mean ± SEM of three independent wells. The significance of these data were analysed by using an unpaired two-sided Student’s t-test. The significance was defined as P < 0.05 (*). The EC_50_ values were calculated using GraphPad Prism (GraphPad Software, Inc., California, USA).

## Additional Information

**How to cite this article**: Fujii, S. *et al.* PGE_2_ is a direct and robust mediator of anion/fluid secretion by human intestinal epithelial cells. *Sci. Rep.* **6**, 36795; doi: 10.1038/srep36795 (2016).

**Publisher’s note:** Springer Nature remains neutral with regard to jurisdictional claims in published maps and institutional affiliations.

## Supplementary Material

Supplementary Information

Supplementary Movie 1

Supplementary Movie 2

Supplementary Movie 3

Supplementary Movie 4

Supplementary Movie 5

Supplementary Movie 6

Supplementary Movie 7

Supplementary Movie 8

## References

[b1] Field M. Intestinal ion transport and the pathophysiology of diarrhea. J. Clin. Invest. 111, 931–943 (2003). 12671039 10.1172/JCI18326 PMC152597

[b2] Welsh M. J., Smith P. L., Fromm M. & Frizzell R. A. Crypts are the site of intestinal fluid and electrolyte secretion. Science 218, 1219–1221 (1982). 6293054 10.1126/science.6293054

[b3] Clevers H. & Batlle E. SnapShot: The Intestinal Crypt. Cell 152, 1198–1198 e2 (2013). 23452862 10.1016/j.cell.2013.02.030

[b4] Barrett K. E. & Keely S. J. Chloride secretion by the intestinal epithelium: molecular basis and regulatory aspects. Annu. Rev. Physiol. 62, 535–572 (2000). 10845102 10.1146/annurev.physiol.62.1.535

[b5] Greger R. Role of CFTR in the colon. Annu. Rev. Physiol. 62, 467–491 (2000). 10845099 10.1146/annurev.physiol.62.1.467

[b6] Park H. W. *et al.* Dynamic regulation of CFTR bicarbonate permeability by [Cl^−^]_i_ and its role in pancreatic bicarbonate secretion. Gastroenterology 139, 620–631 (2010). 20398666 10.1053/j.gastro.2010.04.004

[b7] Berg J., Yang H. & Jan L. Y. Ca^2+^-activated Cl^−^ channels at a glance. J. Cell. Sci. 125, 1367–1371 (2012). 22526416 10.1242/jcs.093260 PMC3336373

[b8] Matthews J. B. Molecular regulation of Na^+^-K^+^-2Cl^−^ cotransporter (NKCC1) and epithelial chloride secretion. World J Surg . 26, 826–830 (2002). 11948363 10.1007/s00268-002-4059-z

[b9] Thiagarajah J. R. & Verkman A. S. Chloride channel-targeted therapy for secretory diarrheas. Current Opinion in Pharmacology 13, 888–894 (2013). 23992767 10.1016/j.coph.2013.08.005 PMC3890324

[b10] Schwartz C. J., Kimberg D. V., Sheerin H. E., Field M. & Said S. I. Vasoactive intestinal peptide stimulation of adenylate cyclase and active electrolyte secretion in intestinal mucosa. J. Clin. Invest. 54, 536–544 (1974). 4369434 10.1172/JCI107790 PMC301586

[b11] Tapper E. J., Powell D. W. & Morris S. M. Cholinergic-adrenergic interactions on intestinal ion transport. Am. J. Physiol. 235, E402–E409 (1978). 211861 10.1152/ajpendo.1978.235.4.E402

[b12] Cooke H. J., Wang Y. Z., Frieling T. & Wood J. D. Neural 5-hydroxytryptamine receptors regulate chloride secretion in guinea pig distal colon. Am. J. Physiol. 261, G833–G840 (1991). 1951703 10.1152/ajpgi.1991.261.5.G833

[b13] Homaidan F. R., Tripodi J., Zhao L. & Burakoff R. Regulation of ion transport by histamine in mouse cecum. European Journal of Pharmacology 331, 199–204 (1997). 9274980 10.1016/s0014-2999(97)00184-2

[b14] Baird A. W., Skelly M. M., O’Donoghue D. P., Barrett K. E. & Keely S. J. Bradykinin regulates human colonic ion transport *in vitro*. British Journal of Pharmacology 155, 558–566 (2008). 18604228 10.1038/bjp.2008.288 PMC2579663

[b15] Kimberg D. V., Field M., Johnson J., Henderson A. & Gershon E. Stimulation of intestinal mucosal adenyl cyclase by cholera enterotoxin and prostaglandins. J. Clin. Invest. 50, 1218–1230 (1971). 4325309 10.1172/JCI106599 PMC292051

[b16] Field M., Musch M. W., Miller R. L. & Goetzl E. J. Regulation of epithelial electrolyte transport by metabolites of arachidonic acid. J. Allergy Clin. Immunol. 74, 382–385 (1984). 6432879 10.1016/0091-6749(84)90135-0

[b17] Hirokawa M. Low-dose PGE_2_ mimics the duodenal secretory response to luminal acid in mice. AJP: Gastrointestinal and Liver Physiology 286, G891–G898 (2004). 10.1152/ajpgi.00458.2003 14764447

[b18] Garcia M. A. S., Yang N. & Quinton P. M. Normal mouse intestinal mucus release requires cystic fibrosis transmembrane regulator-dependent bicarbonate secretion. J. Clin. Invest. 119, 2613–2622 (2009). 19726884 10.1172/JCI38662 PMC2735925

[b19] Karaki S.-I. & Kuwahara A. Regulation of intestinal secretion involved in the interaction between neurotransmitters and prostaglandin E_2_. Neurogastroenterol. Motil. 16 Suppl 1, 96–99 (2004). 15066012 10.1111/j.1743-3150.2004.00482.x

[b20] Fan Y. Y., Davidson L. A., Callaway E. S., Goldsby J. S. & Chapkin R. S. Differential effects of 2- and 3-series E-prostaglandins on *in vitro* expansion of Lgr5^+^ colonic stem cells. Carcinogenesis 35, 606–612 (2014). 24336194 10.1093/carcin/bgt412 PMC3941743

[b21] Fordham R. P. *et al.* Transplantation of expanded fetal intestinal progenitors contributes to colon regeneration after injury. Cell Stem Cell 13, 734–744 (2013). 24139758 10.1016/j.stem.2013.09.015 PMC3858813

[b22] Kaltoft N. *et al.* Prostaglandin E_2_-induced colonic secretion in patients with and without colorectal neoplasia. BMC Gastroenterol . 10, 9–9 (2010). 20100359 10.1186/1471-230X-10-9 PMC2824707

[b23] Evans T. Fishing for a WNT-PGE_2_ link: beta-catenin is caught in the stem cell net-work. Cell Stem Cell 4, 280–282 (2009). 19341616 10.1016/j.stem.2009.03.006

[b24] Sharon P., Ligumsky M., Rachmilewitz D. & Zor U. Role of prostaglandins in ulcerative colitis. Enhanced production during active disease and inhibition by sulfasalazine. Gastroenterology 75, 638–640 (1978). 30669

[b25] Ahrenstedt O., Hällgren R. & Knutson L. Jejunal release of prostaglandin E_2_ in Crohn’s disease: relation to disease activity and first-degree relatives. J. Gastroenterol. Hepatol. 9, 539–543 (1994). 7865710 10.1111/j.1440-1746.1994.tb01557.x

[b26] Rampton D. S. & Sladen G. E. Relationship between rectal mucosal prostaglandin production and water and electrolyte transport in ulcerative colitis. Digestion 30, 13–22 (1984). 6149163 10.1159/000199086

[b27] Sato T., Clevers H. & Clevers H. Growing self-organizing mini-guts from a single intestinal stem cell: mechanism and applications. Science 340, 1190–1194 (2013). 23744940 10.1126/science.1234852

[b28] Sato T. *et al.* Long-term expansion of epithelial organoids from human colon, adenoma, adenocarcinoma, and Barrett’s epithelium. Gastroenterology 141, 1762–1772 (2011). 21889923 10.1053/j.gastro.2011.07.050

[b29] Yui S. *et al.* Functional engraftment of colon epithelium expanded *in vitro* from a single adult Lgr5^+^ stem cell. Nat. Med . 18, 618–623 (2012). 22406745 10.1038/nm.2695

[b30] Mizutani T. *et al.* Real-time analysis of P-glycoprotein-mediated drug transport across primary intestinal epithelium three-dimensionally cultured *in vitro*. Biochem. Biophys. Res. Commun. 419, 238–243 (2012). 22342245 10.1016/j.bbrc.2012.01.155

[b31] Farin H. F. *et al.* Paneth cell extrusion and release of antimicrobial products is directly controlled by immune cell-derived IFN. Journal of Experimental Medicine 211, 1393–1405 (2014). 24980747 10.1084/jem.20130753 PMC4076587

[b32] Dekkers J. F. *et al.* A functional CFTR assay using primary cystic fibrosis intestinal organoids. Nat. Med . 19, 939–945 (2013). 23727931 10.1038/nm.3201

[b33] Schwank G. *et al.* Functional repair of CFTR by CRISPR/Cas9 in intestinal stem cell organoids of cystic fibrosis patients. Cell Stem Cell 13, 653–658 (2013). 24315439 10.1016/j.stem.2013.11.002

[b34] Matsumoto T. *et al.* Increase of bone marrow-derived secretory lineage epithelial cells during regeneration in the human intestine. Gastroenterology 128, 1851–1867 (2005). 15940621 10.1053/j.gastro.2005.03.085

[b35] Foulke-Abel J. *et al.* Human Enteroids as a Model of Upper Small Intestinal Ion Transport Physiology and Pathophysiology. Gastroenterology 150, 638–649 (2016). 26677983 10.1053/j.gastro.2015.11.047 PMC4766025

[b36] Mohajer B. & Ma T. Y. Eicosanoids and the small intestine. Prostaglandins and Other Lipid Mediators 61, 125–143 (2000). 10867125 10.1016/s0090-6980(00)00068-x

[b37] Carty E., De Brabander M., Feakins R. M. & Rampton D. S. Measurement of *in vivo* rectal mucosal cytokine and eicosanoid production in ulcerative colitis using filter paper. Gut 46, 487–492 (2000). 10716677 10.1136/gut.46.4.487 PMC1727893

[b38] Mroz M. S. & Keely S. J. Epidermal growth factor chronically upregulates Ca^2+^-dependent Cl^−^ conductance and TMEM16A expression in intestinal epithelial cells. *J. Physiol.* (*Lond.*) 590, 1907–1920 (2012). 22351639 10.1113/jphysiol.2011.226126 PMC3573312

[b39] Rampton D. S., Sladen G. E. & Youlten L. J. Rectal mucosal prostaglandin E_2_ release and its relation to disease activity, electrical potential difference, and treatment in ulcerative colitis. Gut 21, 591–596 (1980). 7429322 10.1136/gut.21.7.591 PMC1419888

[b40] Schmidt C., Baumeister B., Kipnowski J., Schiermeyer-Dunkhase B. & Vetter H. Alteration of prostaglandin E_2_ and leukotriene B_4_ synthesis in chronic inflammatory bowel disease. Hepatogastroenterology 43, 1508–1512 (1996). 8975956

[b41] Murano T. *et al.* Hes1 promotes the IL-22-mediated antimicrobial response by enhancing STAT3-dependent transcription in human intestinal epithelial cells. Biochem. Biophys. Res. Commun. 443, 840–846 (2014). 24342613 10.1016/j.bbrc.2013.12.061

[b42] Magalhães D., Cabral J. M., Soares-da-Silva P. & Magro F. The role of epithelial ion transports in inflammatory bowel disease. Am. J. Physiol. Gastrointest. Liver Physiol. 310, G460–G476 (2014). 10.1152/ajpgi.00369.2015 26744474

[b43] Ghishan F. K. & Kiela P. R. Epithelial transport in inflammatory bowel diseases. Inflamm. Bowel Dis. 20, 1099–1109 (2014). 24691115 10.1097/MIB.0000000000000029 PMC4103619

[b44] Zachos N. C. *et al.* Human Enteroids/Colonoids and Intestinal Organoids Functionally Recapitulate Normal Intestinal Physiology and Pathophysiology. J. Biol. Chem. 291, 3759–3766 (2016). 26677228 10.1074/jbc.R114.635995 PMC4759158

[b45] Dey I., Lejeune M. & Chadee K. Prostaglandin E_2_ receptor distribution and function in the gastrointestinal tract. British Journal of Pharmacology 149, 611–623 (2006). 17016496 10.1038/sj.bjp.0706923 PMC2014644

[b46] Takafuji V., Cosme R., Lublin D., Lynch K. & Roche J. K. Prostanoid receptors in intestinal epithelium: selective expression, function, and change with inflammation. *Prostaglandins, Leukotrienes and Essential Fatty Acids* (*PLEFA*) 63, 223–235 (2000). 10.1054/plef.2000.0144 11049698

[b47] Preston P. *et al.* Disruption of the K^+^ Channel β -Subunit KCNE3 Reveals an Important Role in Intestinal and Tracheal Cl^−^ Transport. Journal of Biological Chemistry 285, 7165–7175 (2010). 20051516 10.1074/jbc.M109.047829 PMC2844166

[b48] Ito G. *et al.* Lineage-specific expression of bestrophin-2 and bestrophin-4 in human intestinal epithelial cells. PLoS One 8, e79693 (2013). 24223998 10.1371/journal.pone.0079693 PMC3818177

[b49] Oh U. & Jung J. Cellular functions of TMEM16/anoctamin. Pflugers Arch . 468, 443–453 (2016). 26811235 10.1007/s00424-016-1790-0 PMC4751194

[b50] Singh R. D. *et al.* Ano1, a Ca^2+^-activated Cl^−^ channel, coordinates contractility in mouse intestine by Ca^2+^ transient coordination between interstitial cells of Cajal. *J. Physiol.* (*Lond.*) 592, 4051–4068 (2014). 25063822 10.1113/jphysiol.2014.277152 PMC4198014

[b51] Okamoto R. & Watanabe M. Role of epithelial cells in the pathogenesis and treatment of inflammatory bowel disease. J. Gastroenterol. 51, 11–21 (2015). 26138071 10.1007/s00535-015-1098-4

[b52] Medani M. *et al.* Prostaglandin D_2_ regulates human colonic ion transport via the DP1 receptor. Life Sci . 122, 87–91 (2015). 25534438 10.1016/j.lfs.2014.12.017

[b53] Carulli A. J. *et al.* Notch receptor regulation of intestinal stem cell homeostasis and crypt regeneration. Dev. Biol. 402, 98–108 (2015). 25835502 10.1016/j.ydbio.2015.03.012 PMC4433599

[b54] Painter R. G. *et al.* The role of chloride anion and CFTR in killing of Pseudomonas aeruginosa by normal and CF neutrophils. J. Leukoc. Biol. 83, 1345–1353 (2008). 18353929 10.1189/jlb.0907658 PMC2901559

[b55] Church J. A change from HCO_3_^−^-CO_2_^−^ to hepes-buffered medium modifies membrane properties of rat CA1 pyramidal neurones *in vitro*. *J. Physiol.* (*Lond.*) 455, 51–71 (1992). 1336555 10.1113/jphysiol.1992.sp019290 PMC1175633

[b56] Okamoto R. *et al.* Requirement of Notch activation during regeneration of the intestinal epithelia. Am. J. Physiol. Gastrointest. Liver Physiol. 296, G23–G35 (2009). 19023031 10.1152/ajpgi.90225.2008

