# Supplementary material for: PGE2 is a direct and robust mediator of anion/fluid secretion by human intestinal epithelial cells
Source: Sci Rep. 2016 Nov 9;6:36795. doi: 10.1038/srep36795 (PMC5101536; doi:10.1038/srep36795)
Supplement: Supplementary Information [file srep36795-s1.pdf]

PGE_2 is a direct and robust mediator of anion/fluid secretion by human intestinal epithelial cells.

Satoru Fujii, Kohei Suzuki, Ami Kawamoto, Fumiaki Ishibashi, Toru Nakata, Tatsuro Murano, Go Ito, Hiromichi Shimizu, Tomohiro Mizutani, Shigeru Oshima, Kiichiro Tsuchiya, Tetsuya Nakamura, Akihiro Araki, Kazuo Ohtsuka, Ryuichi Okamoto and Mamoru Watanabe

Supplementary Fig. S1

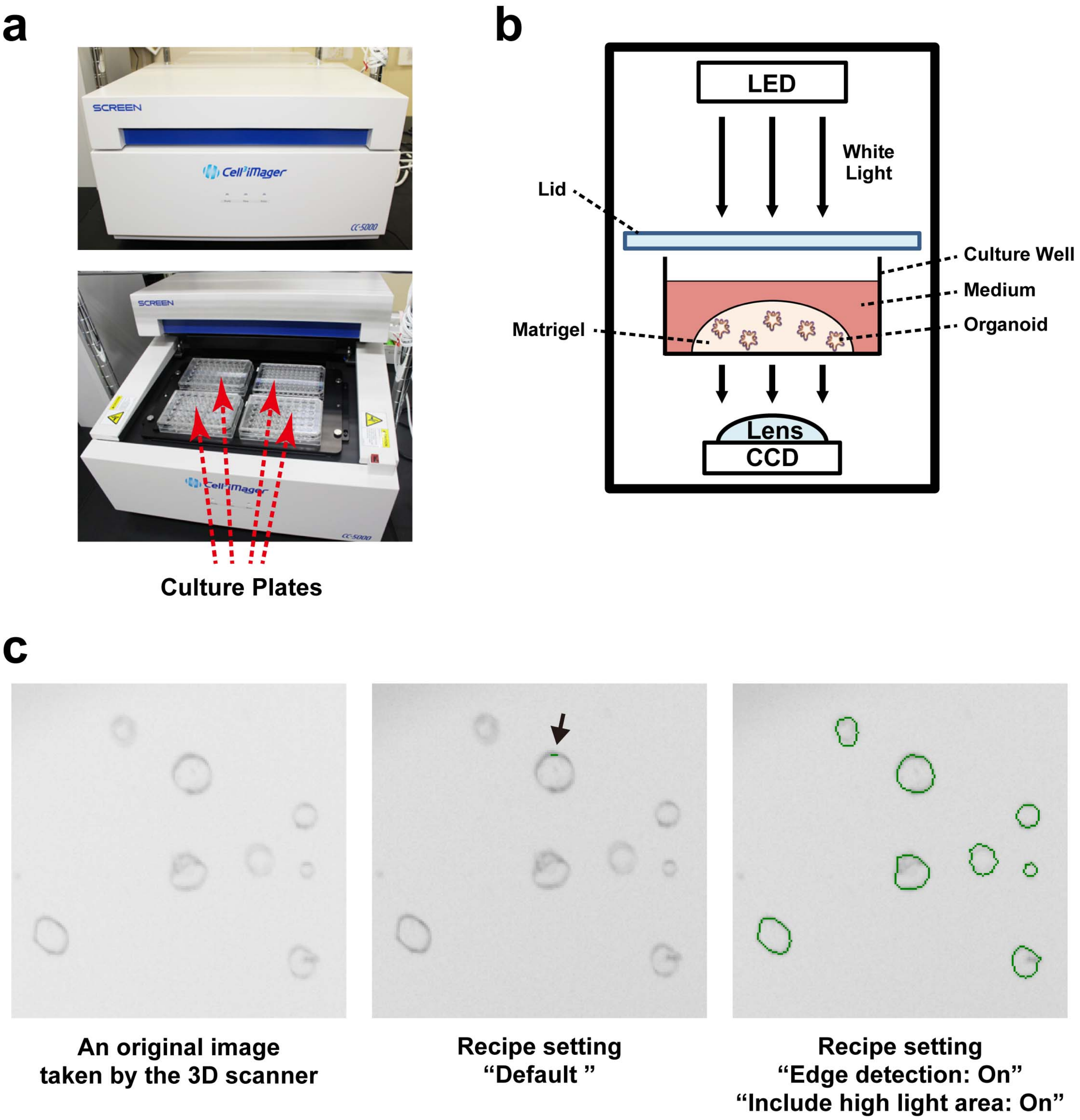

Supplementary Figure S1. System overview of the 3D-scanning system.
(a) An overview of our 3D-scanning system. A maximum of 4 multi-well plates can be loaded onto the stage. Permission was granted by SCREEN Holdings Co., Ltd. to use the logos and trademarks on the equipment. (b) Scheme of the optical detection system is shown. A LED white light device is fitted above the culture plates and a CCD camera is equipped underneath the culture plates. The system can acquire a full-scan image of a single culture plate at a resolution of 4800 dpi within 1 min. (c) The effect of optimizing the image analysis paremeters (recipe) is shown. Only a limitied partial edge (black arrow) could be recognized (green line) by the “default” setting (middle panel) without any adjustment. Turning on the “Edge detection” mode and also the “Include high light area” mode greatly improves the detection of the organoid rim (green line). For details of the parameters, please also refer to Supplementary Table S1.

Supplementary Fig. S2

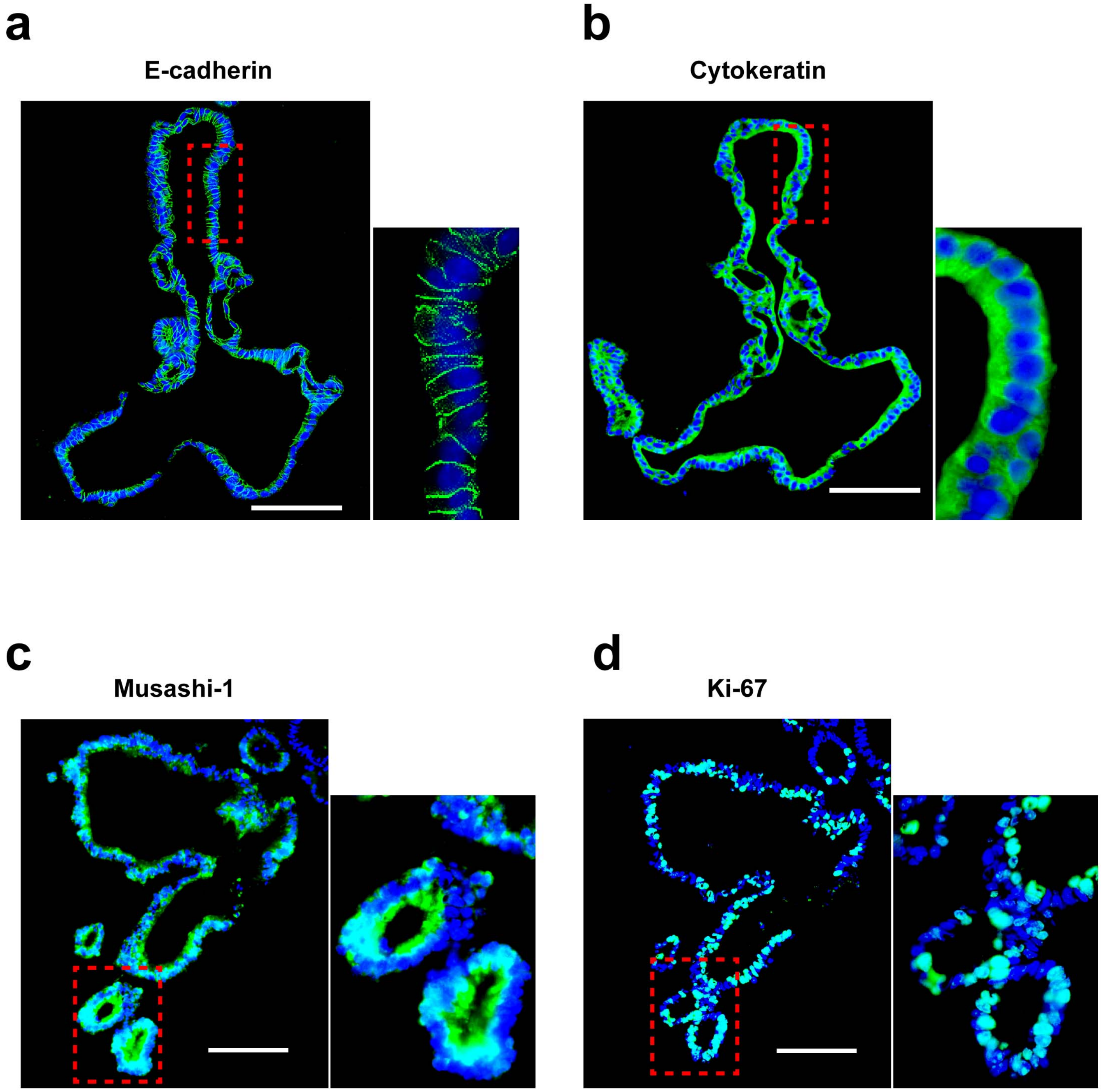

Supplementary Figure S2. Characteristics of the cells composing human small intestinal organoids. Immunostaining of E-cadherin (a) and pan-cytokeratin (b) shows positive staining (green) in all of the cells, confirming that those cells are of epithelial origin. Also, a magnified view of the area designated by the red dotted line confirms the basolateral expression of E-cadherin, indicating that these cells maintain their polarity. Also, immunostaining of the stem cell marker Musashi-1 (c) and the proliferative cell marker Ki-67 (d) shows a broad expression throughout the organoid (green), suggesting that the organoids dominantly consist of stem/progenitor cells. A magnified view of the area designated by the red dotted line is shown at the right side. Scale bar represents 100 µm.

Supplementary Fig. S3

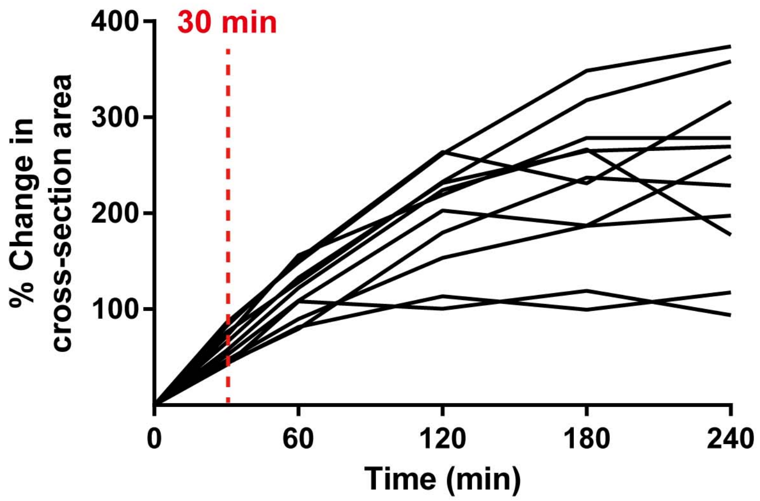

Supplementary Figure S3. Long-term pulse-chase experiment of the forskolin-induced swelling using human small intestinal organoids. Organoid swelling was induced by adding forskolin (10^{-6} M) at the next day of passage, and quantified the swelling response for up to 240 min. Each line represents the response of individual organoids. Note that at time points longer than 30 min, the linear increase in the % change in cross-section area is not necessarily maintained. In the later time period, some organoids show a decrease in their cross-section area, whereas others reach to a plateau. The data were acquired and analyzed based on the time-lapse imaging shown in Supplementary Movie S2 (for up to 240 min), using Image J.

Supplementary Fig. S4

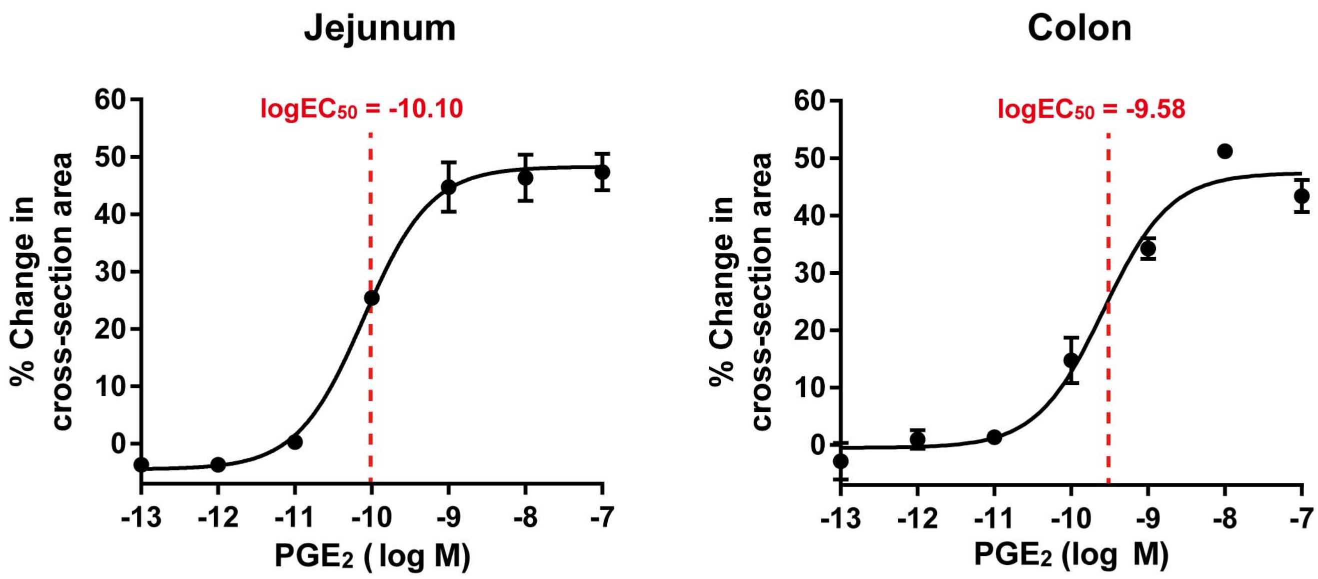

Supplementary Figure S4. Dose response curve of PGE_2-induced swelling using the jejunal and the colonic organoids of a single patient. Another set of data of the dose response curve of PGE_2-induced organoid swelling acquired from human jejunum-derived and colon-derived organoids. These organoids were established from biopsy specimens that were taken from the jejunum and the ascending colon of a single CD patient.

Supplementary Fig. S5

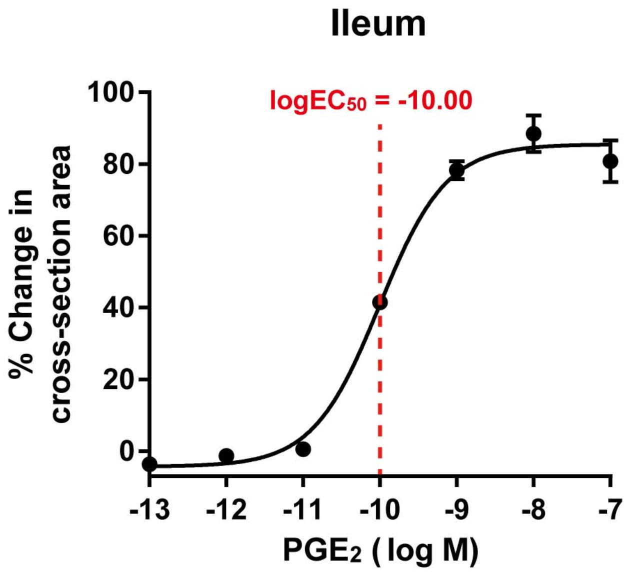

Supplementary Figure S5. Dose response curve of PGE_2-induced swelling using the ileal organoids of an UC patient. A dose response curve of PGE_2-induced organoid swelling was acquired from ileal organoids of an UC patient. Note that the $logEC_{50}$ value is virtually the same as those shown in Fig. 3a.

Supplementary Fig. S6

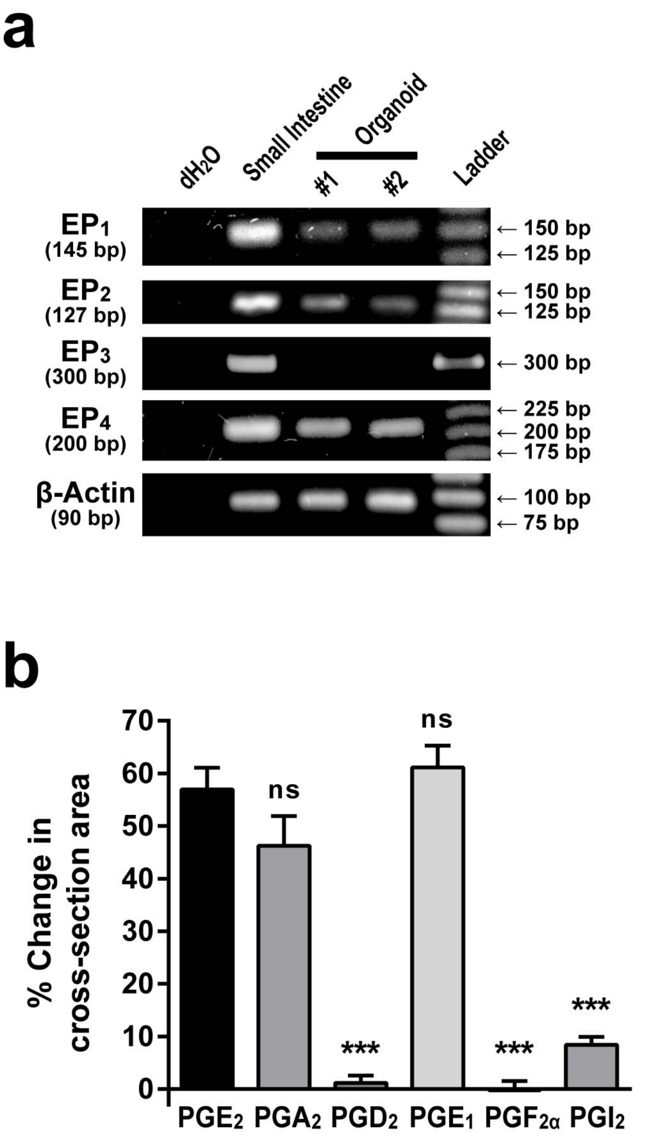

Supplementary Figure S6. Expression of EP receptors in human small intestinal organoids, and their response to other prostaglandins. (a) A semi-quantitative RT-PCR analysis of human small intestinal organoids is shown. Expression of EP_1, EP_2 and EP_4 was confirmed in the subject organoids. However, expression of EP_3 mRNA was below the detection level in the organoids. Results acquired from the whole small intestinal tissue sample are shown as a reference. dH_2O served as a negative control. The number of bp indicates the length of the targeted amplification region for each gene. (b) Induction of human jejunal organoid swelling by 6 different prostaglandins was examined. The concentration of all the prostaglandins was set to 10^{-9} M. Data are shown as the mean±SEM of three independent wells. *** indicates $P<0.0005$, **** indicates $P<0.0001$ as determined by two-sided Student's t-test compared to the data of PGE_2. ns indicates not significant.

Supplementary Fig. S7

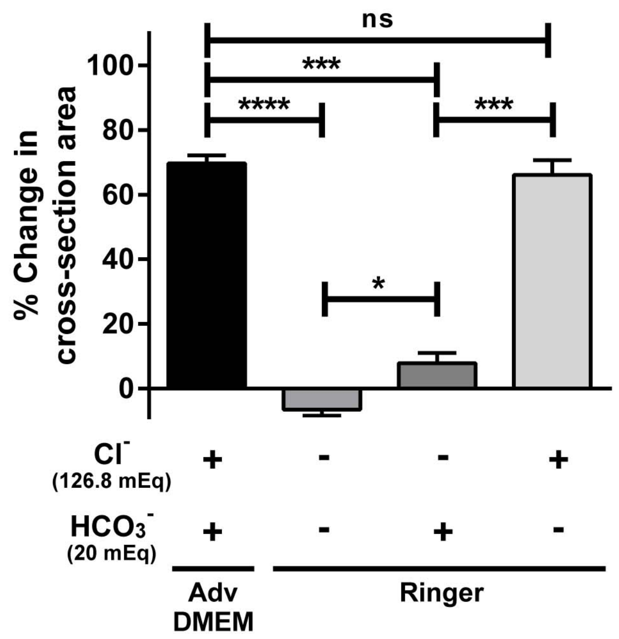

Supplementary Figure S7. Contribution of HCO_3^- or Cl^- to the PGE_2-induced swelling. PGE_2-induced swelling (10^{-9} M) of human jejunal organoids was tested under the depletion of Cl^- alone, HCO_3^- alone or both Cl^- and HCO_3^-. Depletion of both Cl^- and HCO_3^- completely abrogated the swelling response. Supplementation of Cl^- (126.8 mEq) almost completely recovered the swelling response, whereas supplementation of HCO_3^- (20 mEq) partly recovered the swelling response up to only a minimal extent. Data are shown as the mean±SEM of three independent wells. * indicates P<0.05, *** indicates P<0.0005, **** indicates P<0.0001 as determined by two-sided Student's t-test compared between the designated columns. ns indicates not significant.

Supplementary Fig. S8

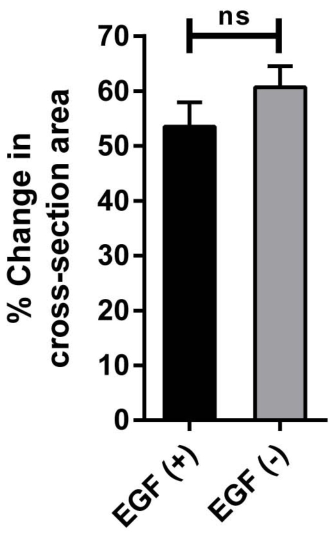

Supplementary Figure S8. Effect of EGF depletion on the PGE_2-induced swelling.
Human jejunal organoids were subjected to PGE_2-induced swelling (10^{-9} M) under normal culture condition (EGF(+)) or without EGF (EGF(-)). Depletion of EGF was started 24 h prior to the addition of PGE_2. Data are shown as the mean±SEM of three independent wells. ns indicates not significant as determined by two-sided Student's t-test.

Supplementary Movie S1. Time-lapse imaging of a jejunal organoid swelling induced by forskolin (10^{-5} M) for up to 60 min at 10 days after passage. Scale bar: 1000 μm.

Supplementary Movie S2. Time-lapse imaging of a jejunal organoid swelling induced by forskolin (10^{-6} M) for up to 360 min at the next day of passage. Scale bar: 100 μm.

Supplementary Movie S3. Time-lapse imaging of a jejunal organoid swelling induced by PGE_2 (10^{-6} M) at 3 days after passage. Scale bar: 100 μm.

Supplementary Movie S4. Time-lapse imaging of a jejunal organoid swelling induced by VIP (10^{-6} M) at 3 days after passage. Scale bar: 100 μm.

Supplementary Movie S5. Time-lapse imaging of a jejunal organoid swelling induced by ACh (10^{-3} M) at 3 days after passage. Scale bar: 100 μm.

Supplementary Movie S6. Time-lapse imaging of a jejunal organoid swelling induced by Histamine (10^{-3} M) at 3 days after passage. Scale bar: 100 μm.

Supplementary Movie S7. Time-lapse imaging of a jejunal organoid swelling induced by Serotonin (10^{-3} M) at 3 days after passage. Scale bar: 100 μm.

Supplementary Movie S8. Time-lapse imaging of a jejunal organoid swelling induced by Bradykinin (10^{-5} M) at 3 days after passage. Scale bar: 100 μm.

Supplementary Table S1. Details of the image analysis settings

Parameters	Function	Recipe settings		
		Adjusted value	Default value	Range of adjustment
Allowable object's maximum area (%)	Maximum size of the object relative to the well size.	25	40	0.1-100
Debris threshold (%)	Threshold to delete an object as a debris, judged by the difference in intensity relative to the background.	25	100	0-100
Compactness upper limit	Threshold to delete an object whose value of circumference^2 / (4π x Area) is above the set number.	200	300	0-500
Edge detection	Select to use the edge detection mode or not. (Also "Include high light area" is set to "on")	On	Off	On or Off
Edge candidate	Edge intensity below this value is ignored.	20	Off	Off or 1-120
Edge threshold	Edge intensity over this value is recognized as the true edge.	75	Off	Off or 0-120
Object edge smoothing	Select to use smoothing of the edge mode or not.	On	Off	On or Off
Spheroid size lower limit (μm^2)	Minimum area size of an organoid that should be subjected to analysis.	1500	0	
Spheroid size upper limit (μm^2)	Maximum area size of an organoid that should be subjected to analysis.	2400000	2147483647	
Spheroid volume lower limit	Minimum suspected volume size of an organoid that should be subjected to analysis.	0	0	
Spheroid volume upper limit	Maximum suspected volume size of an organoid that should be subjected to analysis.	2400000	2147483647	
Circularity lower limit (%)	Object whose value of (4π x Area) / circumference^2 is lower than the set limit is ignored.	45	0	0-100
Sharpness lower limit	Object with a sharpness value below the set limit is ignored.	0	1	0-100
Spheroid density upper limit	Maximum mean optical density value of the subject organoids.	30	300	0-400

Supplementary Table S2

Composition of normal, Cl^--free, and HCO_3^--supplemented Cl^--free Ringer's solutions

	Ringer's solution	Cl^--free Ringer's solution	HCO_3^--supplemented Cl^--free Ringer's solution
NaCl	122 mM	None	None
$MgCl_2$	1.2 mM	None	None
$CaCl_2$	1.2 mM	None	None
K_2HPO_4	2.4 mM	2.4 mM	2.4 mM
KH_2PO_4	0.6 mM	0.6 mM	0.6 mM
HEPES	20 mM	20 mM	None
Glucose	10 mM	10 mM	10 mM
Na gluconate	None	122 mM	122 mM
Mg gluconate	None	1.2 mM	1.2 mM
Ca gluconate	None	4 mM	4 mM
$NaHCO_3$	None	None	20 mM
pH	7.3	7.3	7.3
